# Recombinant activated factor VII in controlling bleeding in non-hemophiliac patients

**DOI:** 10.4103/0256-4947.62830

**Published:** 2010

**Authors:** Farjah H. AlGahtani, Mashael Alshaikh, AbdulRehman AlDiab

**Affiliations:** a From the Division of Hematology, Department of Medicine, King Saud University, Riyadh, Saudi Arabia; b From the Department of Pharmacy, King Saud University, Riyadh, Saudi Arabia

## Abstract

**BACKGROUND:**

There have been recent reports on the successful use of recombinant factor VIIa (rFVIIa) in non-hemophiliac patients who have experienced heavy blood loss due to trauma with extensive organ damage and who have received multiple blood transfusions with hemostatic changes without success. The timing of administration, dosage, mortality, units of blood transfusion saved, risk of thrombotic events, and the risk/benefit ratio are still poorly defined.

**PATIENTS AND METHODS:**

We conducted a retrospective review of all medical records of patients who received rFVIIa between January 2003 and March 2008. Data collection included demographic characteristics, diagnosis, indications, comorbidities, and amount of blood products used with rFVIIa, dose of rFVIIa, mortality, and adverse events.

**RESULTS:**

We identified 45 patients, 27 (60%) males and 18 (40%) females, with a median age of 52 years. The median dose of rFVIIa was 40 μg/kg (range, 20-120 μg/kg). Five (11.1%) patients needed a second dose of rFVIIa (dose range of 20-85 μg/kg) whereas three patients (6.7%) needed a third dose of rFVIIa (dose range of 40-60 μg/kg). There was a marked and significant reduction in transfusion requirements for packed red blood cells (*P*=.0078). Overall transfusion requirements significantly decreased after the infusion of rFVIIa (*P*=.0323). Nineteen patients (42.2%) died and thrombosis was documented in 3 patients (6.7%).

**CONCLUSION:**

Use of rFVIIa should be based on sound clinical evidence to balance the risks, benefits, and cost if used among non-hemophiliacs. Prospective randomized studies are needed to investigate the efficacy and cost-effectiveness of rFVIIa for this indication and to allow a final assessment of the importance of this treatment.

Recombinant factor VIIa (rFVIIa, Novo Seven, Novo Nordisk, Denmark) is a vitamin K-dependent glycoprotein that is an analog of the naturally occurring protease. In 1999, the US Food and Drug Administration approved its use for the treatment of bleeding episodes in patients with hemophilia and in patients with factor VIII or factor IX inhibitors.^1^–^3^ The success of rFVIIa in controlling hemophiliac bleedings has led to its use for other serious bleeding episodes, such as in non-hemophiliac patients with active bleeding and as a prophylactic agent for high-risk patients with obstetrical bleeding,^4^–^8^ hemorrhage associated with warfarin toxicity,^9^,^10^ intracerebral hemorrhage,^11^–^12^ upper gastrointestinal bleeding,^13^,^14^ intractable bleeding after cardiopulmonary bypass in children and adults and Glanzmann's thrombocytopenia.^15^–^18^

Several studies have reported the “off-label” use of rFVIIa.^5^–^8^,^10^–^15^,^17^,^18^ Only two randomized, placebo-controlled, double-blind trials were conducted to evaluate the efficacy and safety of rFVIIa among trauma patients with massive bleeding.^19^ The rest are case reports and studies in a small number of subjects. Due to its mechanism of action, rFVIIa can lead to thrombotic complications such as myocardial and cerebral ischemia,^20^,^21^ deep venous thrombosis or pulmonary thromboembolism.^10^,^15^,^22^,^23^ The rate of thromboembolic adverse events (TAEs) related to the use of rFVIIa in hemophiliacs has been 5% to 7% since its introduction.^4^,^9^,^24^–^29^ This is largely attributed to its action on activated platelets at sites of bleeding only, and the lack of a systemic effect. A recent review of submissions to the US FDA Adverse Event Reporting System suggested that the risk of TAE and death in nonhemophiliac recipients of rFVIIa was 7% to 15%.^30^ Adverse events reported to the FDA suggested that most thromboembolic events were associated with the use of higher doses of rFVIIa and for “off-label” indications, resulting in serious morbidity and mortality.^30^

Early experience and dose finding trials of rFVIIa have suggested that a dose of 90 to 120 μg/kg confers adequate hemostatic effect for most surgical and non-surgical bleeding.^27^ Doses of 60 to 90 μg/kg have been used with efficacy between 80% and 87% in serious bleeds and 91% and 94% in surgical bleeding.^26^ Incremental doses of rFVIIa have been investigated by various workers to determine if such a strategy leads to better control of bleeding with 71% to 84% effective response.^24^,^26^,^28^ The data on megadosing, however, need to be investigated further. Pre-existing risk factors for thromboembolic events were strongly associated with adverse events reported with the use of higher doses of rFVIIa and for “off-label” indications. In the most compelling evidence of possible thromboembolic risk associated with rFVIIa, serious thromboembolic event rates of 7% (mainly myocardial and cerebral infarction) were reported in a randomized placebo-controlled study of rFVIIa for intracerebral hemorrhage involving 399 patients.^31^ As larger doses and more intensified regimens are being studied for hemophiliacs, thrombotic risks with rFVIIa need to be better characterized. “Safe” dosing regimen should be well defined, especially among nonhemophiliacs and older patients. At present, high-quality randomized studies on the use of rFVIIa in the setting of uncontrolled massive bleeding remain scarce. Hence, a consensus is needed to guide physicians in their practice to make critical decisions in this regard. The questions still remain: Is rFVIIa safe? How effective is it in controlling massive uncontrolled bleeding? Do the benefits outweigh the risks?

## PATIENTS AND METHODS

We reviewed the records of all nonhemophiliac patients from King Khalid University Hospital, King Saud University, Riyadh, Saudi Arabia who received rFVIIa to control bleeding between January 2003 and March 2008 and were on follow-up for up to 3 months after the infusion of rFVIIa. Patients included in the study were adults (age >17 years) who had bleeding that could not be controlled by conventional transfusion therapy. Data collection included demographic characteristics (age, sex, weight), diagnosis, co-morbidities, cause of bleeding, mortality, cause of death, dose of rFVIIa, medication (anticoagulation or antiplatelets before bleeding), transfusion data (amount of RBCs, fresh-frozen plasma [FFP], platelets, cryoprecipitate before and up to 48 hours after infusion of rFVIIa), and surgical data (type and indication of operation, re-operation and during operation). Adverse events such as TAEs were recorded. Ethical approval was obtained from the Ethics Committee of King Saud University College of Medicine and Research Center, Riyadh, Saudi Arabia prior to the conduct of the study.

Statistical analysis was done with the Statistical Package for Social Sciences volume 16.0 (SPSS Inc., Chicago, Illinois, USA). Demographic characteristics and baseline characteristics are expressed as median values and ranges. The Mann-Whitney test was used to evaluate the differences and compare transfusion data before and after administration of rFVIIa. The Pearson correlation was used for association between variables. Significance was assumed at *P*<.05.

## RESULTS

Of 65 patients who received rFVIIa from January 2003 to March 2008, 20 patients were excluded of whom 18 were hemophiliacs and two were children. In total, 45 patients were included in the analysis. Twenty-seven (60%) were male and 18 (40%) female and the median age was 52 years (range, 17-79 years). None of the 45 patients had any of the registered indications for the use of rFVIIa (hemophilia A or B with inhibiting antibodies, FVII deficiency or Glanzmann thrombasthenia). Bleeding was post-CABG (n=28 patients; 62.2%), gastrointestinal tract (n=5, 11.1%), post-partum (n=5, 11.1%), central nervous system (n=3, 6.7%) and other surgical procedures (n=4, 8.9%). The median APACHE score was 10 (range, 2-33) (Table 1).

**Table 1:** Characteristics of rFVIIa use at King Khalid University Hospital, Riyadh.

No. of patients	45
Age, median (range) (years)	52 (17-79)
rFVIIa dose in μg/kg, median (range)	40 (20-120)
APACHE score, median (range)	10 (2-33)
No. of patients who received transfusion prior to rFVIIa	31 (68.9%)
No. of patients who received transfusion after rFVIIa	17 (37.8%)
No. of patients who received additional doses of rFVIIa	8 (17.8%)
No. of patients with control of bleeding	28 (62.2%)
Thrombosis	3 (6.7 %)
Death	19 (42.2%)

The median frequency of rFVIIa administration was two doses per patient (range, 1-4) and the median dose of rFVIIa was 40 μg/kg (range, 20-120 μg/kg). Prior to infusion of rFVIIa, 31 patients (68.9%) were given FFP and, if necessary, platelet transfusion (n=27, 60%), packed red blood cells (PRBC) (n=27, 60%), and cryoprecipitate (n=18, 40%). Bleeding was stopped completely in 28 patients (62.2%) whereas 17 (37.8%) patients needed additional blood products after the initial rFVIIa dose. Five patients needed a second dose of rFVIIa (median dose, 40 μg/kg) and three patients needed a third dose of rFVIIa (median dose, 60 μg/kg).

Transfusion requirements before and in the first 48 hours after the administration of rFVIIa are shown in Table 2. There was a marked reduction in transfusion requirements for PRBCs, from a mean of 9.5 units before rFVIIa to a mean of 5.6 units after rFVIIa. However, no reductions were observed in the transfusion requirements for FFP, platelets, and cryoprecipitate. Overall transfusion requirements decreased after the infusion of rFVIIa.

**Table 2:** Differences in transfusion requirements before and after infusion of rFVIIa.

Transfusions	Mean before rFVIIa	Mean after rFVIIa	Mean difference
No. of units packed red blood cells	9.5	5.6	3.9
No. of units fresh frozen plasma	5.6	5.0	0.6
No. of units platelets	7.1	6.6	0.5
No. of units cryoprecipitate	6.7	4.6	2.1
No. of units for total transfusion	28.9	21.8	7.1

**Figure 1:**
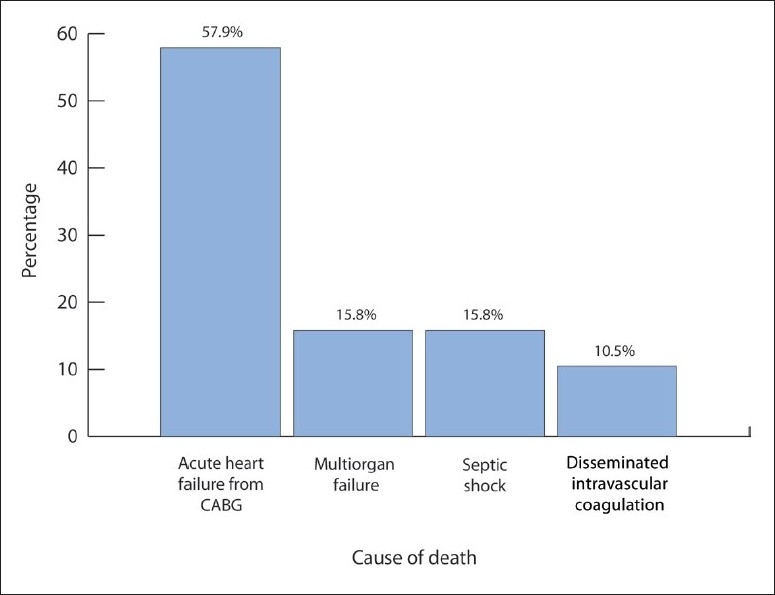
Cause of death among non-hemophiliacs receiving rFVIIa

Nineteen patients (42.2%) died of which 11 (57.9%) were male and eight (42.1%) were female. The median age of the patient population in this study was 51.5 years (range, 17-76 years) and the median APACHE score was 18 (range, 9-33). The median dose of rFVIIa administered was 45 μg/kg (range, 20-120 μg/kg). Thirteen patients (68.4%) were given FFP, 12 (63.2%) were given platelets, 12 (63.2%) were given PRBC, and nine (47.4%) given cryoprecipitate. Eleven patients (57.9%) died of acute heart failure from CABG, three (15.8%) of multi-organ failure, three (15.8%) of septic shock, and two (10.5%) of disseminated intravascular coagulation (Figure 1). Thrombosis was documented in three patients (6.7%): two males and a female. The median age was 49 years (range, 21-60 years). Acute DVT occurred in the right superior femoral and popliteal veins in a patient one week after rFVIIa infusion, two patients had a cerebrovascular accident, one occurred ten days after rFVIIa infusion and the other three weeks after rFVIIa infusion. The median APACHE score was 16.5 (range, 2-19). The cause of bleeding was post-cardiac surgery in all three patients. The median dose of rFVIIa administered was 30 μg /kg (range, 20-40 μg/kg.) All three patients died of acute heart failure post-CABG.

Patients who died had received a higher median dose of rFVIIa (60 μg/kg vs 25 μg/kg) and were older (58 years vs 53 years) than those who lived. Massive bleeding was documented to a greater extent among patients who died despite the transfusion of additional blood products compared to those who survived (26.3% vs 10.0%).

## DISCUSSION

In this study, the mortality rate due to rFVIIa use among non-hemophiliacs was higher than that reported elsewhere.^29^,^30^ This could be explained by the demographic characteristics of our patients (as evidenced by the higher APACHE score of those who died), the inherent impending mortality of the patient's condition, the dose of rFVIIa, the late infusion of rFVIIa, the use of transfusion products, and several other factors such as age, the presence of co-morbid conditions, and electrolyte disorders. Despite higher doses of rFVIIa, massive blood transfusion, and even additional doses of rFVIIa and blood transfusion, bleeding could not be controlled, especially among our patients who have APACHE scores more than 15. However, our study suggests that the infusion of rFVIIa may significantly reduce transfusion requirements and may be beneficial to patients. The transfusion requirement of PRBC and the overall transfusion requirements were significantly reduced in the observation period after rFVIIa administration, compared to before rFVIIa administration by as much as 41% for PRBC and 24.6% for overall transfusion.

Of 19 deaths, 11 (57.9%) were a result of acute heart failure from cardiac surgery. Excessive intraoperative bleeding remains a major complication following cardiac surgery, resulting in increased morbidity and mortality. The massive bleeding associated with the disruption of coagulation mechanisms of the body is such that no amount of blood transfusion can benefit the patient without proper monitoring of the coagulation profile. The principal cause of non-surgical, hemostatic, peri-operative bleeding is a pre-existing, undetected bleeding disorder that could be related to the nature of the operation itself or that arises from coagulation abnormalities due to massive blood loss. Despite several reports claiming the benefits of using rFVIIa in post-operative cardiac surgery bleeding,^15^–^18^ most are case reports or reports from small number of subjects, and hence, the risks and benefits of rFVIIa remain unclear.

Our rate of thrombotic events was 6.7% is in accordance with a 2006 report^30^ that stated that most of the thromboembolic AEs follow the unlabeled use of rFVIIa. Our results are in accordance with the recent meta-analysis report by Hsia^29^ who reported that mortality was 15% among 3184 nonhemophiliac patients and 7.8% had TAE. Our study showed 42.2% mortality and a 6.7% incidence of TAE.^15^,^18^ Very often these adverse events and mortality are due to the combination and coexistence of various pathologies. Identifying patients at risk remains a major component of preventing excessive blood loss. Understanding the hemostatic changes occurring in the intra- and peri-operative period, especially in complex procedures like cardiopulmonary bypass and orthotopic liver transplantation, is crucial in developing new strategies for the management of peri-operative bleeding. This is alarming in the sense that almost half of our patients died despite 68.9% of our patients receiving additional blood products prior to the administration of rFVIIa and even higher doses of rFVIIa.

The effectiveness of rFVIIa in controlling bleeding in 28 of our patients and the observed mortality in 19 patients may have been influenced by the timing of dosing, the interval of administration, and the underlying cause of bleeding. As this was not a controlled trial, we cannot be certain that the reduction in transfusion requirements can be attributed to rFVIIa alone as several other factors such as the patient's condition (low APACHE score), age, and even the absence of co-morbid conditions could have an effect on the survival of these patients. The effect of rFVIIa may be enhanced if it is given early in the course of blood loss. Significant delay in the use of rFVIIa can be avoided because a temporary reduction in bleeding does not reduce mortality.^26^,^28^,^32^,^33^ The administration of rFVIIa could potentially lead to thromboembolic events as seen in three of our patients.

These findings lead us to suggest the use of rFVIIa in specific situations such as in younger patients (<50 years old), in patients with low APACHE scores, in patients whose electrolyte imbalances have been corrected, and in patients whose coagulation profiles have been determined and addressed. Inadvertent use of rFVIIa in patients with high APACHE scores (>15), older patients, and in those with co-morbidities, may be futile considering the high cost of the drug and its associated risks and mortality. Prescribing rFVIIa in non-hemophiliacs should be based upon high-quality clinical evidence so as to balance the risks, benefits, and costs. This evidence, however, is scant for clinicians at the bedside or for hospitals that bear the economic consequences of prescriptive decisions. This is more so in a set-up where the guidelines are not formulated nor are the prescriptions of rFVIIa regulated.

Prospective randomized studies are needed to investigate the efficacy, safety and cost-effectiveness of rFVIIa to allow a final assessment of the importance of this treatment for use in non-hemophiliacs. This would help to evaluate the potential hemostatic benefits and adverse effects of rFVIIa. Until prospective, randomized, controlled data are available on the use of rFVIIa, especially in the control of massive bleeding among non-hemophiliacs, we recommend close monitoring of coagulation parameters before, during, and after therapy, especially among high-risk patients (with high APACHE scores, old patients >50 years old, and with significant co-morbidities).
